# Long days enhance recognition memory and increase insulin-like growth factor 2 in the hippocampus

**DOI:** 10.1038/s41598-017-03896-2

**Published:** 2017-06-20

**Authors:** Adriano Dellapolla, Ian Kloehn, Harshida Pancholi, Ben Callif, David Wertz, Kayla E. Rohr, Matthew M. Hurley, Kimberly M. Baker, Samer Hattar, Marieke R. Gilmartin, Jennifer A. Evans

**Affiliations:** 1 Department of Biomedical Sciences, Marquette University, Milwaukee, WI 53233 USA; 2 Departments of Biology & Neuroscience, Johns Hopkins University, Baltimore, MD 21218 USA

## Abstract

Light improves cognitive function in humans; however, the neurobiological mechanisms underlying positive effects of light remain unclear. One obstacle is that most rodent models have employed lighting conditions that cause cognitive deficits rather than improvements. Here we have developed a mouse model where light improves cognitive function, which provides insight into mechanisms underlying positive effects of light. To increase light exposure without eliminating daily rhythms, we exposed mice to either a standard photoperiod or a long day photoperiod. Long days enhanced long-term recognition memory, and this effect was abolished by loss of the photopigment melanopsin. Further, long days markedly altered hippocampal clock function and elevated transcription of Insulin-like Growth Factor2 (*Igf2*). Up-regulation of *Igf2* occurred in tandem with suppression of its transcriptional repressor Wilm’s tumor1. Consistent with molecular de-repression of *Igf2*, IGF2 expression was increased in the hippocampus before and after memory training. Lastly, long days occluded IGF2-induced improvements in recognition memory. Collectively, these results suggest that light changes hippocampal clock function to alter memory, highlighting novel mechanisms that may contribute to the positive effects of light. Furthermore, this study provides insight into how the circadian clock can regulate hippocampus-dependent learning by controlling molecular processes required for memory consolidation.

## Introduction

Light is used to treat psychiatric disease in humans, but the neurobiological basis of how light affects cognitive function remains unclear. For more than three decades, light therapy has been used to treat a variety of mood disorders, including seasonal depression, major depression, postpartum depression, and bipolar disorder^1–4^. In addition, increased light exposure has been shown to delay the onset of dementia and enhance quality of life in patients with Alzheimer’s and Parkinson’s disease^5–7^. Consistent with these positive effects, light can improve arousal and attention in humans^4, 8^. Clinical studies have established the therapeutic potential of light, and increased insight into the underlying mechanisms may further improve treatment efficacy and help to develop pharmacomimetics^9, 10^.

A common finding in clinical studies is that light is most effective when it is timed to influence the phase and/or waveform of daily rhythms^11^. Daily rhythms are regulated by the circadian system, which is a hierarchical collection of biological clocks located throughout the brain and body^12^. At the cellular level, circadian rhythms are generated by a molecular oscillator involving ca. 24 h rhythms in the transcription and translation of “clock genes”^13, 14^. At its core, the molecular circadian clock is sustained by a negative feedback loop with positive elements that serve as activators and negative elements that act as repressors. The positive elements are bHLH-PAS transcription factors CLOCK and BMAL1, which form a dimer that binds at an E-box to initiate transcription of *Period* (*Per1*, *Per2*, *Per3*) and *Cryptochrome* (*Cry1*, *Cry2*) genes. The corresponding protein products (i.e. PER1-3, CRY1-2) form dimers that feed back to inhibit their own expression by antagonizing the transcriptional activity of CLOCK-BMAL1. These negative elements are degraded over time, thus relieving repression and allowing transcription to recommence the following day. In addition to this primary loop, there are a number of interconnected accessory loops that modulate core clock gene expression. For instance, the activator ROR and the repressor REV-ERBα regulate *Clock* and *Bmal1* expression by competing at RRE elements in the promoter sequences of these genes. In addition, the molecular oscillator gates the expression of numerous “clock-controlled genes” to ensure that cellular processes occur at the appropriate time of day.

Several lines of evidence suggest that there are strong connections between neuropsychiatric disease and changes in circadian clock function^15, 16^. Notably, neuropsychiatric symptoms often co-present with disrupted daily rhythms of sleep, arousal, appetite, and hormone release. Rhythm disturbances may even precede onset of the disease, and disrupted rhythms can be alleviated by treatments that influence clock function (e.g., light). Effects of light on cognitive function are thought to be mediated by a specialized visual system^17, 18^, which regulates circadian function and other non-image forming visual processes^19^. In addition to disrupted rhythms in behavior and physiology, daily rhythms of gene expression are altered in the brains of patients with major depression^20^. Further, genome wide association studies have revealed that human polymorphisms in circadian clock genes are associated with disease incidence and/or severity^21, 22^. Lastly, the molecular link between circadian clock function and psychiatric disease is supported by preclinical studies demonstrating that clock gene mutations produce cognitive deficits in animal models^15, 23^. Despite the implications of this cumulative work, the mechanisms connecting circadian genetic programs to cognitive function are not well understood.

One major obstacle to understanding the neurobiological bases of light therapy is the lack of animal models for studying the positive effects of light on cognition^24^. In fact, most rodent models employ lighting conditions that cause cognitive deficits and disrupt circadian timekeeping (reviewed in ref. 25). While these models can reveal how disrupted rhythms influence cognition, they can provide limited insight into the mechanisms underlying beneficial effects of light. In the present study, we employ a mouse model that simulates a protocol shown to be highly efficacious in humans – namely increased exposure to light during the morning hours^26^. Bright light therapy is provided to humans typically using short exposure to high intensity fluorescent light (i.e., 10,000 lux for 30 min). Although this is an aversive stimulus for rodents, the circadian visual system can integrate photons delivered over time^27, 28^. Thus to avoid negative side effects, we employed longer exposure to lower intensity illumination, effectively producing a long day photoperiod in accord with the ecological niche of rodents (i.e., seasonal changes in day length in the wild). In previous work, we have shown that this long day lighting condition does not eliminate daily rhythms or temporal coordination among clocks within the circadian system^29, 30^. In contrast to previous models where light produces cognitive deficits in nocturnal rodents, we find that exposure to this long day condition improves learning and memory in mice. Having developed this mouse model, we investigate the bases of this positive effect of light on brain function and reveal a novel mechanism by which light and the circadian clock regulate cellular processes required for recognition memory.

## Results

**Figure 1:**
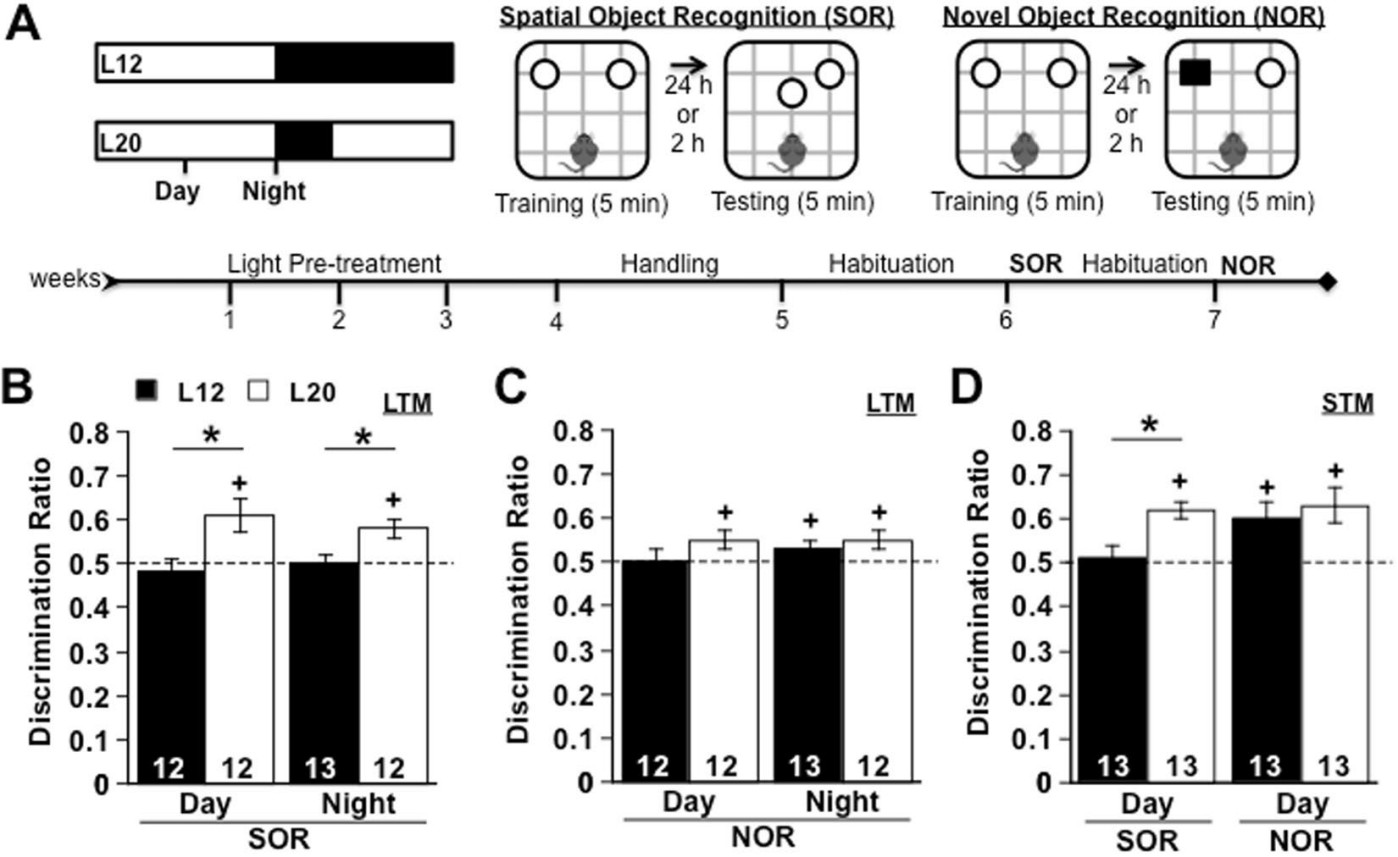
Long days enhance recognition memory. (**A**) Spatial Object Recognition (SOR) and Novel Object Recognition (NOR) memory tested under control (L12) or long day (L20) conditions. Testing for long-term memory (LTM) and short-term memory (STM) occurred 24 h and 2 h after training, respectively. Day = 6 h before lights-off, Night = time of lights-off. Time of training/testing (TT) was selected based on previous work demonstrating that the daily locomotor rhythm is entrained to lights-off in both photoperiodic conditions. (**B**) L20 improved SOR LTM performance during day and night (Full Factorial ANOVA Photoperiod: F(1,1) = 14.35, p < 0.0005, TT: F(1,1) = 0.54, p > 0.4, Photoperiod*TT: F(1,1) = 0.01, p > 0.9). Further, L20 mice performed above chance on the SOR test at both times of day, whereas L12 mice did not (L20 Day: t(11) = 2.39, p < 0.02; L20 Night: t(11) = 4.67, p < 0.0005; L12 Day: t(11) = −0.94, p > 0.8; L12 Night: t(12) = −0.25, p > 0.5). (**C**) L20 mice performed above chance on the NOR test during day and night, whereas L12 mice did so only at night (L20 Day: t(11) = 2.43, p < 0.05; L20 Night: t(11) = 2.24, p < 0.05; L12 Day: t(11) = 0.99, p > 0.1; L12 Night: t(12) = 1.97, p < 0.05). No differences were observed for NOR (Full Factorial ANOVA Photoperiod: F(1,1) = 0.46, p > 0.5, TT: F(1,1) = 0.32, p > 0.5, Photoperiod*TT: F(1,1) = 0.08, p > 0.7). (**D**) L20 mice displayed better STM for SOR (t(24) = −2.33, p < 0.05), but not NOR (t(24) = −0.08, p > 0.9). Number at the base of each bar indicates sample size. *L12 versus L20, two sample Student’s t test, p < 0.05. + Differs from 0.5, one-sample Student’s t test, p < 0.05.

To investigate how light may influence cognitive function, we exposed mice to either 12 h of light/day (L12) or 20 h light/day (L20) and then assessed long-term memory (LTM) using tests for spatial object recognition (SOR) and novel object recognition (NOR), as illustrated in Fig. 1A. Recognition memory was assessed at one of two times of day specifically selected based on the timing of daily rhythms under these photoperiodic conditions^29, 30^ (Fig. 1A). Overall, exposure to long days enhanced memory performance (Fig. 1B), consistent with positive effects of light in healthy humans^8^. Whereas L12 mice failed to discriminate spatial location in the SOR test (p > 0.5), L20 mice displayed a significant preference for the re-located object (p < 0.05). Further, L20 mice displayed better SOR performance than L12 mice both during the day and night (Fig. 1B, p < 0.05), suggesting that memory was increased by L20 rather than merely altered in the timing of peak performance. In addition, L20 mice performed above chance on the NOR test during both daytime and nighttime (Fig. 1C, p < 0.05), but L12 mice only performed above chance during the night (Fig. 1C, p < 0.05) and not during the day (Fig. 1C, p > 0.15). Importantly, no group differences were evident during habituation, object training, or mood tests (Supplementary Figure S1), indicating that long day exposure improved cognitive function without producing anxiety- or depression-like symptoms. Given that SOR is dependent on the hippocampus^31^, this pattern of results suggests that exposure to this long day lighting condition influences hippocampal function.

To define the process by which long days increased cognitive function, we next tested whether memory was enhanced through improved short-term acquisition or long-term consolidation. L12 and L20 mice were trained during the daytime and tested 2 h later to assess short-term memory (STM) performance (Fig. 1D). Even when tested shortly after training, L20 mice performed better than L12 mice on the SOR test (Fig. 1D, p < 0.05), indicating enhancement through improved acquisition. In contrast, both groups performed equally well on the NOR test (Fig. 1D, p > 0.4), suggesting that light modestly improved consolidation for this form of recognition memory. While effects on memory retrieval cannot be completely ruled out in our design, the pattern of results is most consistent with an improvement in memory formation. Again the largest effects were evident for SOR memory, which implicates a change in the function of the hippocampus. To test whether positive effects of light generalized to other hippocampus-dependent memory tasks, mice from both lighting conditions were trained and tested for contextual fear conditioning. In contrast to effects on SOR, L12 mice displayed higher rates of freezing than L20 mice (Supplementary Figure S2, p < 0.05). Interestingly, L20 mice were less sensitive to fear conditioning than L12 mice, with the former group failing to develop high levels of freezing during acquisition like the control group (Supplementary Figure S2). Collectively, these results indicate that light differentially improves learning in a task-specific manner.

**Figure 2:**
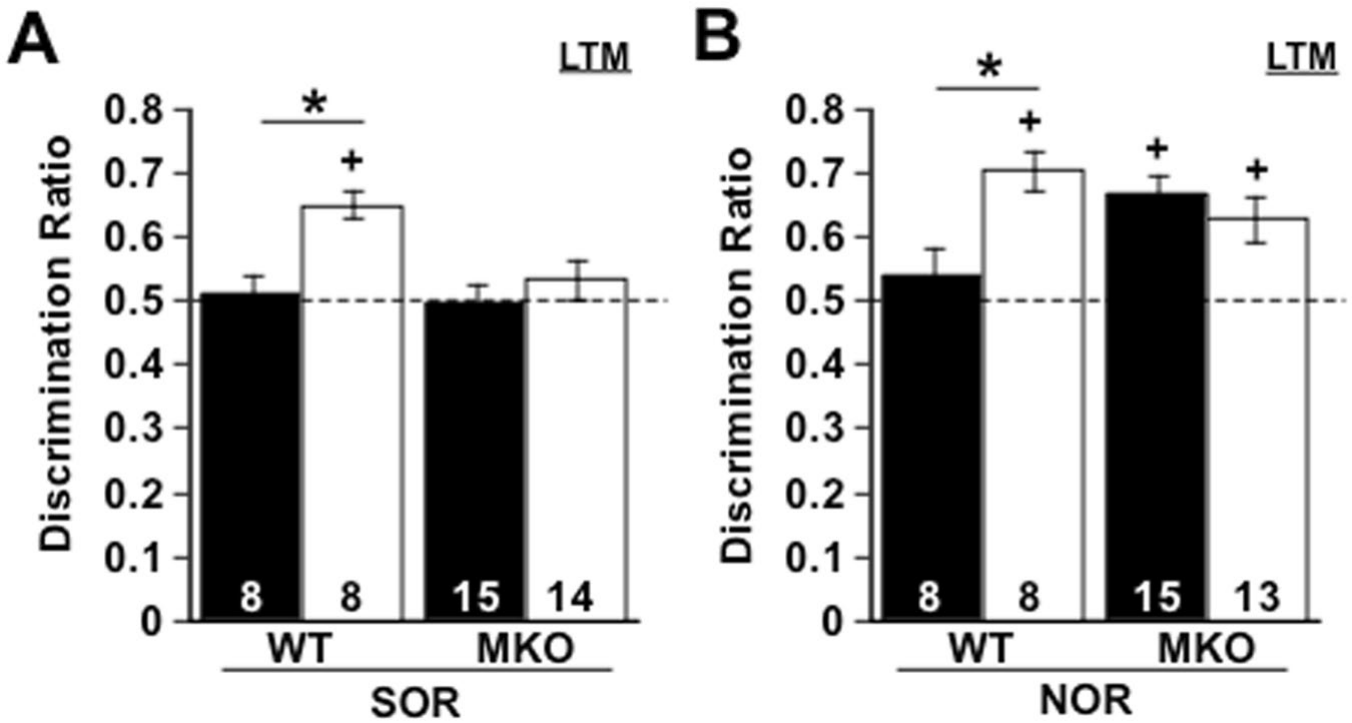
Melanopsin signaling contributes to photoperiodic changes in recognition memory. L20 enhanced Spatial Object Recognition (SOR, **A**) and Novel Object Recognition (NOR, **B**) memory in wild-type mice, but not melanopsin knockout mice (SOR: Full Factorial ANOVA Photoperiod: F(1,1) = 6.55, p < 0.05, Genotype: F(1,1) = 1.27, p > 0.2, Photoperiod*Genotype: F(1,1) = 4.39, p < 0.05; NOR: Full Factorial ANOVA Photoperiod: F(1,1) = 1.56, p > 0.2, Genotype: F(1,1) = 0.04, p > 0.8, Photoperiod*Genotype: F(1,1) = 6.3, p < 0.05). Number at the base of each bar indicates sample size. *L12 versus L20, two sample Student’s t test, p < 0.05. + Differs from 0.5, one-sample Student’s t test, p < 0.05.

In rodent models, loss of melanopsin prevents light from adversely affecting mood and memory^32^. To test the role of this photopigment in mediating positive effects of light on cognitive function, we examined whether loss of melanopsin would prevent long days from enhancing recognition memory. Melanopsin knockout (MKO) and wild-type controls (WT) were housed under L12 or L20, then tested for LTM on both the SOR and NOR memory task. Consistent with our earlier experiments, WT L20 mice performed better than WT L12 mice, with long days significantly enhancing both SOR and NOR memory (Fig. 2, p < 0.05). Notably, the photoperiodic discrepancy in performance was abolished in MKO mice for both SOR and NOR (Fig. 2, p > 0.1), with no difference between MKO and WT mice during habituation or object training (Supplementary Figure S3, p > 0.5). On the SOR test, neither MKO L12 mice nor MKO L20 mice were able to discriminate the object in the novel location (Fig. 2A), suggesting that the loss of melanopsin blocked the positive effect of light. In contrast, both MKO L12 and MKO L20 groups were able to discriminate the novel object on the NOR task (Fig. 2B). Because MKO mice performed above chance on the NOR task (Fig. 2B, p < 0.05), this indicates that visual acuity was not adversely affected by loss of melanopsin. Rather, this pattern of results suggests that the poor NOR performance in WT L12 mice was caused by a visual process that involves melanopsin signaling, which is consistent with previous work using a different mouse model^32^. Taken together, these results indicate melanopsin signaling contributes to both the positive and negative effects of light. Further, the nature of the change induced by loss of melanopsin was task-specific, which may reflect differential roles of distinct centrifugal projections of melanopsin-expressing retinal ganglion cells.

**Figure 3:**
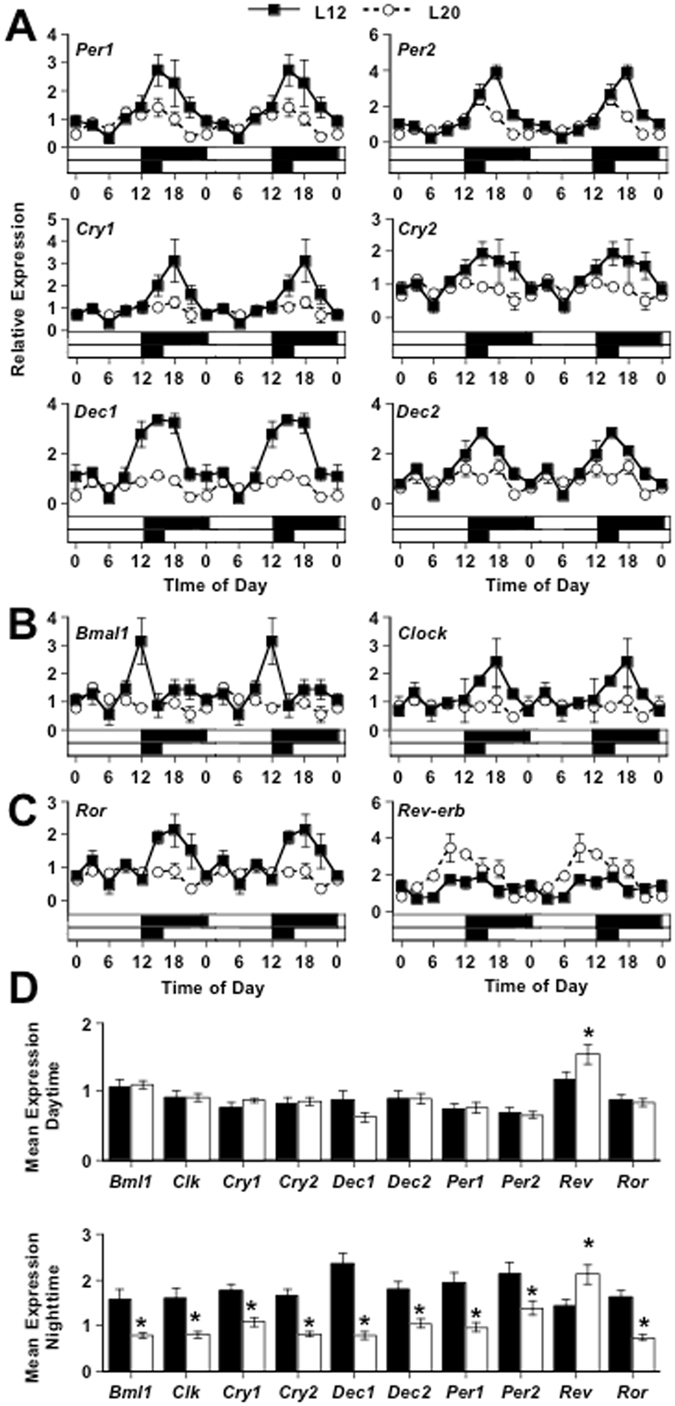
Long days alter clock function in the dorsal hippocampus. L20 attenuated rhythms in most core clock genes (**A,B**) and ancillary clock genes (**C**), with the exception of *Rev-erb*. Note that daily changes in gene expression are double-plotted to facilitate rhythm analysis. (**D**) L20 altered clock gene expression in a phase- and transcript specific manner. *Student’s t-test, p < 0.05. For most clock genes, nighttime expression was decreased, with the exception that *Rev-erb* expression was increased. Statistical evaluation of transcript rhythms and group differences are described in Table S1.

Given the well-described effects of light on daily rhythms, we next postulated that light enhances recognition memory by influencing circadian clock function within the hippocampus. To test this hypothesis, we investigated the effects of long days on daily transcriptional programs in the hippocampus (Fig. 3). Under L12, the hippocampus displayed significant rhythms in a wide variety of circadian genes (Table S1, p < 0.05), which extends previous work on the hippocampal clock^33^ by demonstrating the functional regulation of elements in both core (i.e., *Per1, Per2, Cry1, Cry2, Clock, Bmal1*) and ancillary loops (i.e., *Dec1*, *Dec2, Ror, Rev-erbα*). Notably, nearly all these daily rhythms were attenuated or completely abrogated by L20 (Fig. 3, Table S1). For example, L20 reduced the amplitude of daily rhythms in *Per1, Per2, Cry1*, and *Cry2* by lowering expression specifically during the night (Fig. 3A,D, Table S1, p < 0.05), consistent with our previous work^30^. Likewise, the expression of *Dec1* and *Dec2* was attenuated under L20 (Fig. 3A,D, Table S1, p < 0.05), revealing that light induces widespread suppression of transcripts regulated by *Clock* and *Bmal1*. Consistent with decreased levels of *Clock-Bmal1* regulated transcripts, L20 lowered daily expression of both *Clock* and *Bmal1* (Fig. 3B,D Table S1, p < 0.05). These results indicate that exposure to long days markedly suppresses core clock function in the hippocampus.

To test the molecular drivers underlying light-driven suppression of hippocampal core clock function, we next analyzed daily expression of *Ror* and *Rev-erbα* (Fig. 3C). Notably, we find that long day exposure altered both *Ror* and *Rev-erbα* expression (Fig. 3C), exerting opposite changes in the expression of each transcript. Under L20, *Ror* expression was suppressed during the night similar to the core elements of the molecular feedback loop (Fig. 3C,D, Table S1, p < 0.05). In contrast, L20 up-regulated transcription of *Rev-erbα*, increasing the amplitude of daily rhythms by increasing expression during the late day and early night (Fig. 3C,D, Table S1, p < 0.05). This reveals that not all molecular clock elements are suppressed by light, and suggests that attenuation of core clock function in the hippocampus may be caused by up-regulation of the transcriptional repressor *Rev-erbα*.

**Figure 4:**
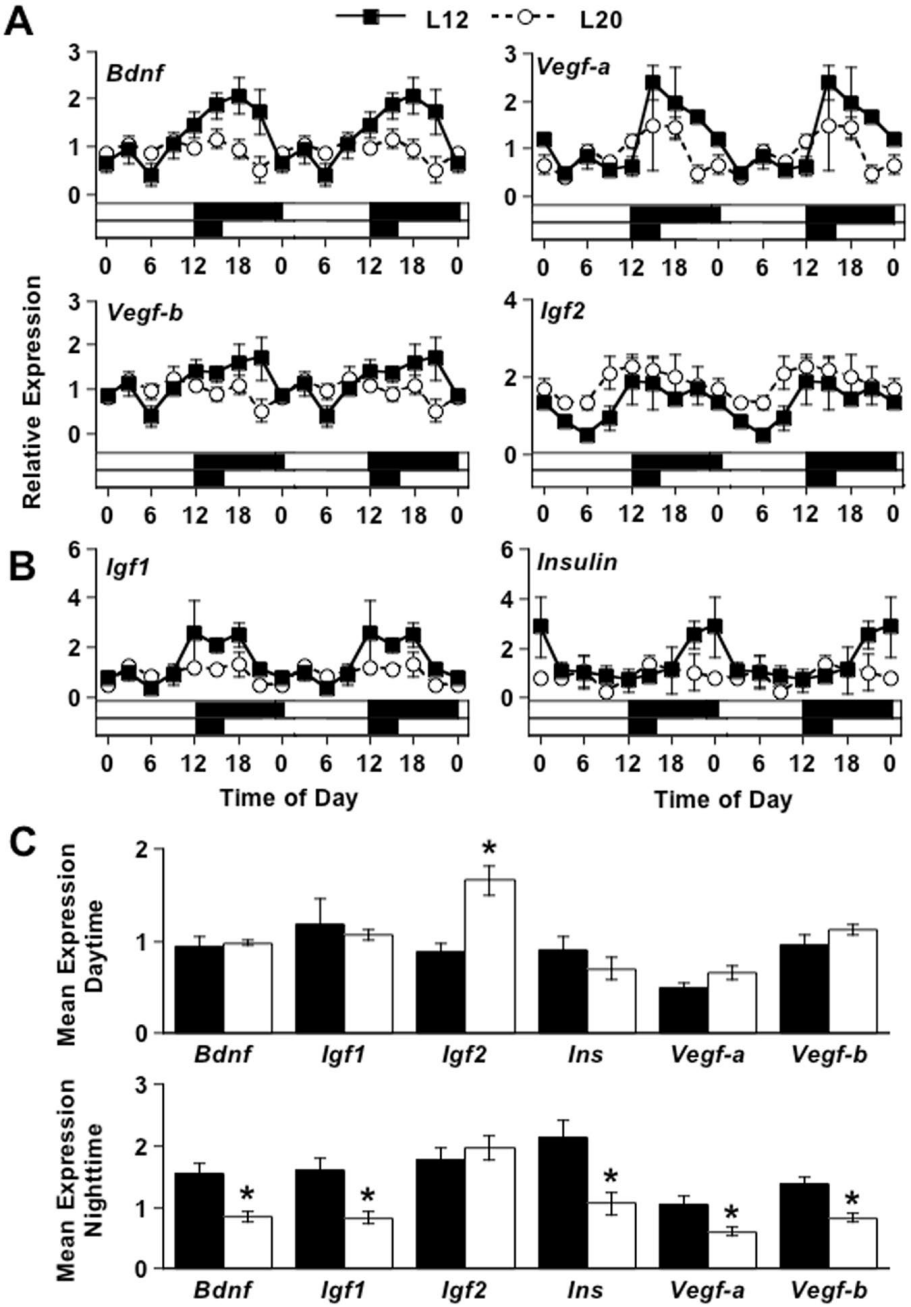
Long days influence hippocampal expression of growth factors in a transcript-specific manner. (**A**) L20 decreased *Bdnf*, *Vegf-a*, and *Vegf-b*, but increased daytime levels of *Igf2*. (**B**) L20 did not elevate *Igf1* or *Insulin*. (**A**,**B**) Note that daily changes in gene expression are double-plotted to facilitate rhythm analysis. Statistical evaluation of transcript rhythms and group differences are described in Table S1. (**C**) Summary of results illustrating that L20 suppressed transcription for most growth factors during the night, but L20 increased *Igf2* expression specifically during the daytime. *Student’s t-test, p < 0.05.

To examine how photic changes in the molecular clock could enhance recognition memory, we next tested the extent to which long days influenced daily expression of growth factors implicated in hippocampus-dependent memory^34^. Under L12, daily fluctuations were observed in each growth factor examined (i.e., *Bdnf, Vegf, Insulin, Igf1, Igf2*), which demonstrates daily regulation of a wide variety of transcripts implicated in memory consolidation (Fig. 4A, Table S1). Moreover, the level of expression for each transcript increased just prior to night (Figs 4A,3C), which is the time of peak performance for many hippocampus-dependent memory tasks in nocturnal rodents^35^. Given this temporal profile, it is likely that circadian gating of growth factor expression contributes to daily rhythms in memory performance. Next, we assessed how growth factor rhythms were altered by L20. Interestingly, the daily expression of nearly all transcripts was suppressed (e.g., *Bdnf*, Fig. 4A, Table S1) with one exception: *Igf2*, which was elevated specifically at the end of the day (Fig. 4A,C). Notably, L20 did not increase expression of *Igf1* or *Insulin* (Fig. 4B,C, Table S1), suggesting that the effects of light were specific to *Igf2*. Given that IGF2 improves hippocampus-dependent memory^36–38^, this suggests that up-regulation of this specific transcript may contribute to light-induced enhancement of cognitive function.

**Figure 5:**
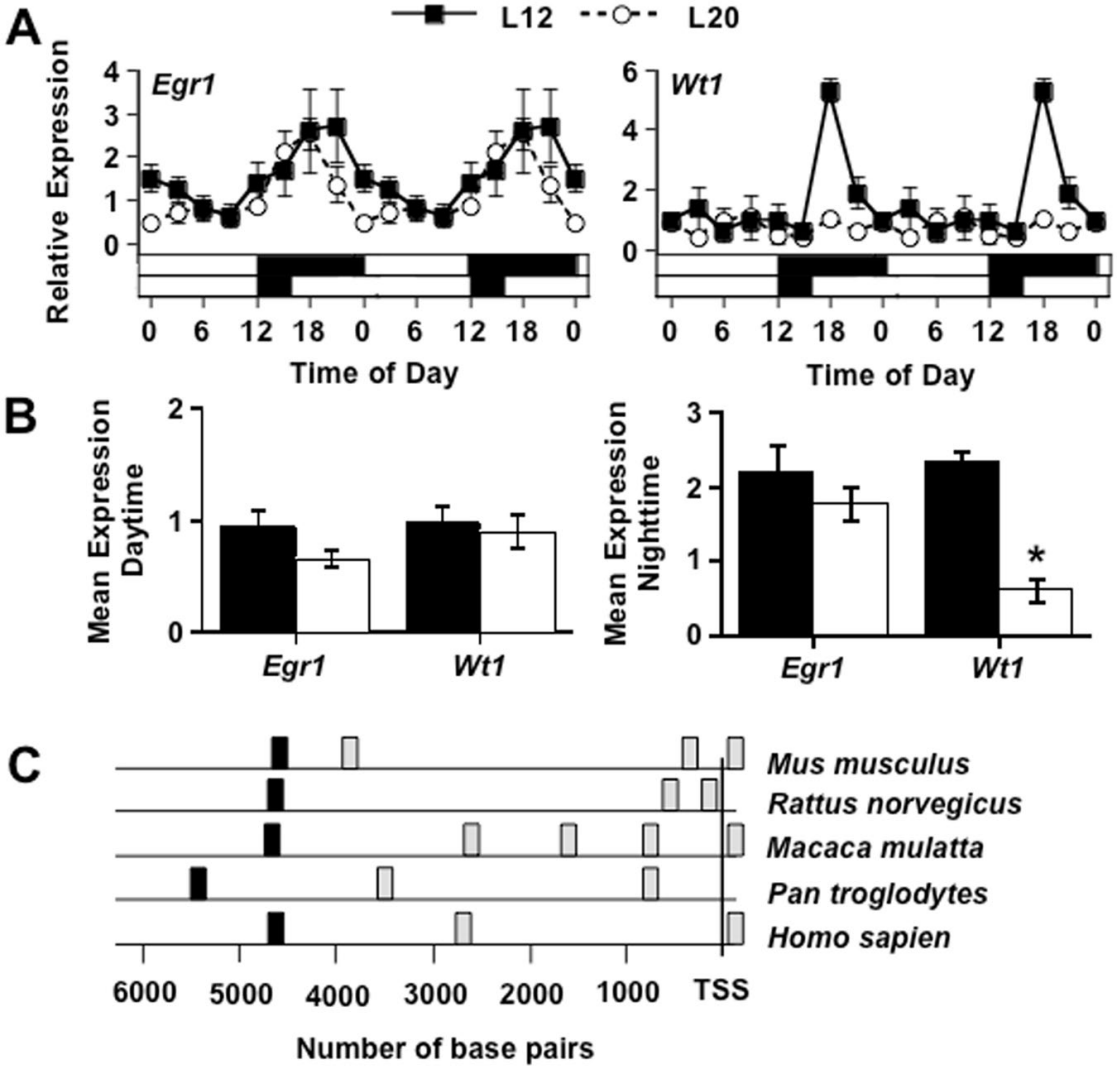
Long days suppress expression of a transcriptional repressor of *Igf2* known as Wilm’s tumor1(*﻿Wt1)*. (**A**) L20 decreased the rhythmic expression of *Wt1*, but produced only a modest effect on *Erg1*. Note that daily changes in gene expression are double-plotted to facilitate rhythm analysis. Statistical evaluation of transcript rhythms and group differences are described in Table S1. (**B**) Summary of results illustrating that *Wt1* expression is reduced at night. (**C**) Sites in the promoter region of *Wt1* that contain an E-box (gray, CA(C/T)GT(G/T)) or an RRE (black, AATCTGGGGTCA) in five different mammalian species. TSS = transcriptional start site.

Based on these results, we predicted that light altered *Igf2* transcription by either increasing its activation and/or decreasing its repression. To examine the molecular mechanisms linking the circadian clock to *Igf2* expression, we assessed daily and light-induced changes in *Igf2* regulators – namely the activator *Egr1* (Early growth response protein1) and the repressor *Wt1* (Wilm’s Tumor1). In the L12 hippocampus, daily rhythms in both *Egr1* and *Wt1* were evident (Fig. 5A, Table S1), with the profile of the rhythm consistent with the distinct role of each transcript in modulating *Igf2* expression. In particular, peak *Wt1* expression occurred at midnight (Fig. 5A), which precedes the time when *Igf2* levels start to fall in the hippocampus (c.f., Fig. 4A). Under L20, *Wt1* expression was completely suppressed, whereas *Egr1* was only modestly affected (Fig. 5A,B, Table S1). This pattern of results indicates that increased daytime transcription of *Igf2* may occur by loss of *Wt1* repression. Interestingly, the time of peak *Wt1* expression under L12 occurred at a phase similar to *Per, Cry*, and *Dec* (c.f., Fig. 3A), which suggests direct regulation of *Wt1* transcription by CLOCK and BMAL1. Further, the waveform of the *Wt1* rhythm mimicked that of *Bmal1* (c.f., Fig. 3B), suggesting that *Wt1* is regulated by other circadian regulatory elements (i.e., RRE^39^). Consistent with both of these predictions, the sequence upstream of the transcription start site of *Wt1* contains both E-box and RRE elements (Fig. 5C). Thus, light-induced suppression of *Wt1* expression may be due to down-regulation of CLOCK/BMAL1 and/or up-regulation of REV-ERBα. Further, these regulatory sequences were observed in rodents, non-human apes, and humans (Fig. 5C), suggesting that circadian regulation of *Wt1* may be conserved across a variety of species. Collectively, this pattern of results indicates that up-regulation of *Igf2* under L20 is likely driven by light-induced changes in molecular clock function that lead to loss of daily *Wt1* repression.

**Figure 6:**
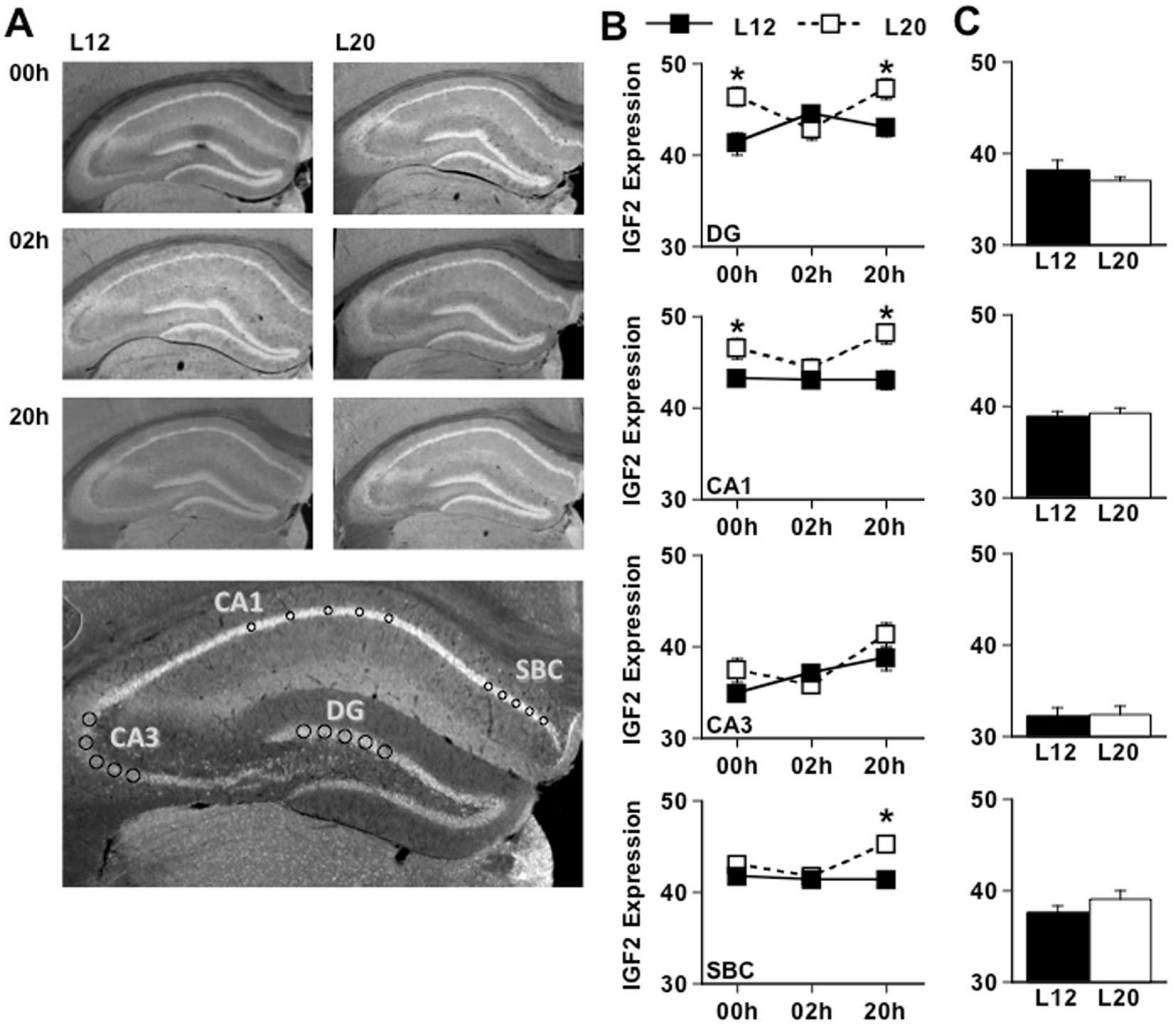
Long days increase IGF2 expression in the dorsal hippocampus before and after SOR training. (**A**) Representative images illustrating changes in IGF2 expression caused by long day exposure and SOR training. To measure IGF2 expression, fluorescence intensity was averaged across five regions of interest placed within the Dentate Gyrus (DG), Cornu Ammonis 1 (CA1), Cornu Ammonis 3 (CA3), and Subiculum (SBC). (**B**) L20 altered IGF2 expression in specific hippocampal subregions (Full Factorial ANOVA DG- Photoperiod: F(1,90) = 9.11, p < 0.01, Time: F(2,90) = 1.18, p > 0.3, Photoperiod*Time: F(2,90) = 6.13, p < 0.01; CA1- Photoperiod: F(1,90) = 19.63, p < 0.0001, Time: F(2,90) = 2.31, p > 0.1, Photoperiod*Time: F(2,90) = 1.95, p > 0.1; CA3- Photoperiod: F(1,90) = 0.14, p > 0.1, Time: F(2,90) = 7.95, p < 0.01, Photoperiod*Time: F(2,90) = 2.20, p > 0.1; SBC- Photoperiod: F(1,90) = 9.31, p < 0.01, Time: F(2,90) = 2.92, p = 0.06, Photoperiod*Time: F(2,90) = 3.17, p < 0.05. * L12 versus L20, LS Means Contrasts, p < 0.05. **C**. IGF2 expression in the hippocampus did not differ between untrained L12 and L20 mice analyzed at the time equivalent to 20 h after SOR training (Student’s t test DG- t(1,30) = 0.68, p > 0.5; CA1- t(1,30) = −1.79, p > 0.05; CA3- t(1,30) = −0.24, p > 0.5; SBC- t(1,30) = −1.23, p > 0.2).

Given the link between IGF2 and hippocampus-dependent memory^36–38^, we next tested whether L20 increased IGF2 expression under basal conditions and/or after recognition memory training (Fig. 6A). First, we examined whether daytime up-regulation of *Igf2* under L20 translated to higher protein expression by examining IGF2 levels prior to SOR training (i.e., 6 h before lights-off, referred to as 00 h in Fig. 6). Consistent with increased *Igf2* transcription under L20, long days elevated daytime expression of IGF2 in the dentate gyrus and CA1 regions of the hippocampus (Fig. 6B, p < 0.05), which may prime the hippocampus to enhance memory acquisition and consolidation. Next, we examined IGF2 expression after SOR training to investigate changes during short-term (2 h) and long-term (20 h) memory processing (Fig. 6A). Because IGF2 in the rat is up-regulated 20 h after training but not 2 h after training^38^, we postulated that L20 may elevate IGF2 levels 20 h after training. Consistent with this hypothesis, L20 increased IGF2 levels 20 h after training (Fig. 6B, p < 0.05), but not 2 h after training (Fig. 6B, p > 0.1). Importantly, time-matched controls did not differ in IGF2 expression, suggesting that training itself caused the photoperiodic difference to emerge after 20 h (Fig. 6C, p > 0.1). Further evidence for training-induced effects was that IGF2 was elevated in the subiculum of L20 mice 20 h after training but not at 00 h (Fig. 6B). These results indicate that increased hippocampal expression of IGF2 is a molecular signature of long days, which may underlie its beneficial effects on cognitive function.

**Figure 7:**
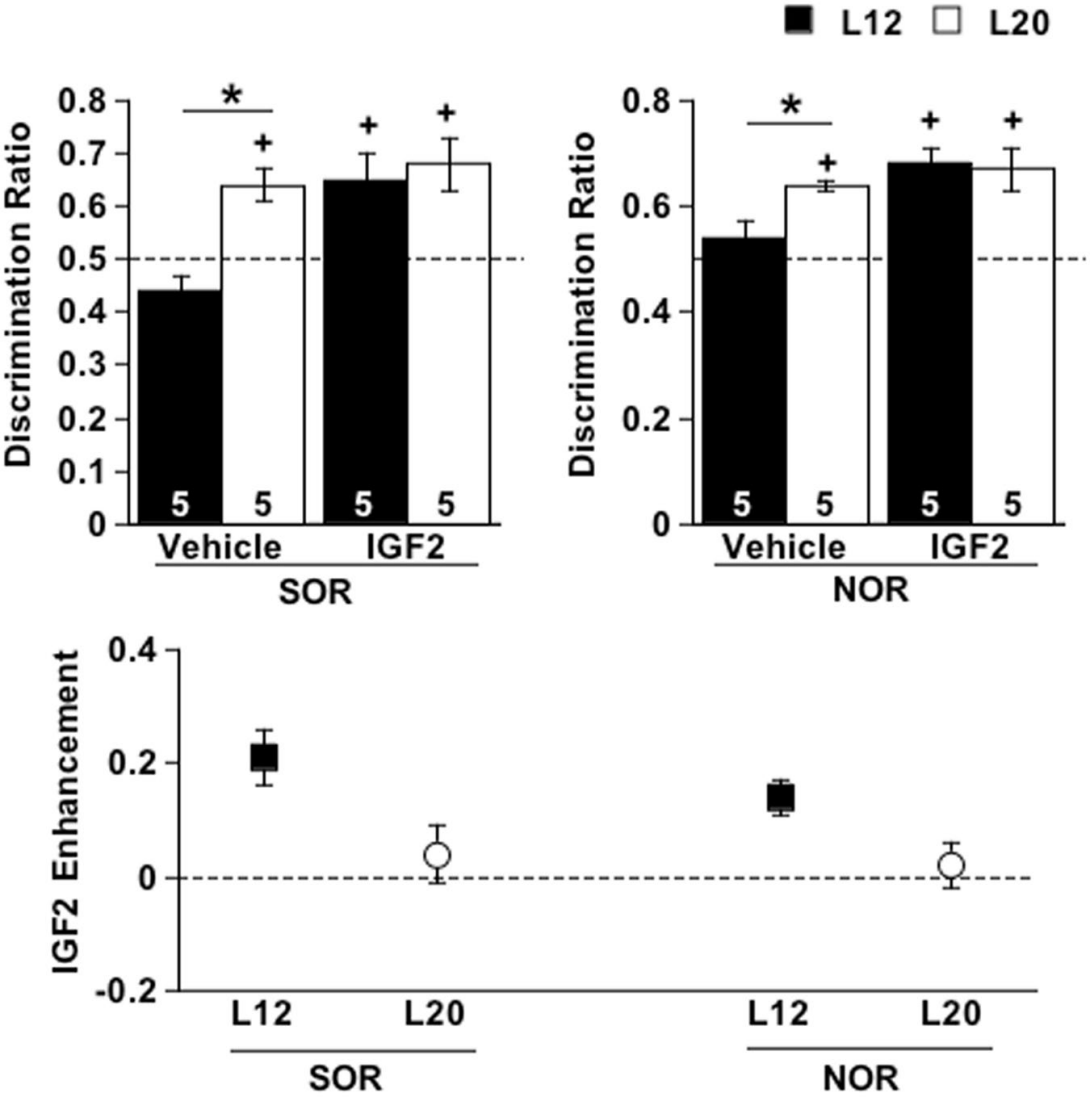
Long days occlude the memory-enhancing effect of IGF2. IGF2 improved SOR and NOR memory in L12 mice, but not L20 mice (Full Factorial ANOVA Photoperiod: F(1,1) = 2.18, p > 0.1, IGF2: F(1,1) = 7.78, p < 0.05, Photoperiod*IGF2: F(1,1) = 4.25, p < 0.05). *L12 versus L20, LS Means Contrasts, p < 0.05. + Differs from 0.5, one-sample Student’s t test, p < 0.05.

Last, to test whether light enhances memory by increasing IGF2, we examined whether long days would occlude the memory-enhancing effects of IGF2 when mice were tested during the daytime. Previous work demonstrates that systemic injections of IGF2 given 20 min prior to training increase hippocampus-dependent memory consolidation in mice^36^. Based on the hypothesis that increased expression of IGF2 underlies L20-induced memory enhancement, we predicted that IGF2 injections would increase memory performance in L12 mice, but not L20 mice. In vehicle-treated mice, long days enhanced SOR and NOR performance (Fig. 7, p < 0.05), which indicates that the photoperiodic enhancement of memory was not adversely influenced by injections. Consistent with previous work, L12 mice given IGF2 injections 20 min prior to object training (Supplementary Figure S4) improved SOR and NOR memory by 15–20% (Fig. 7, p < 0.005). In contrast, L20 mice given IGF2 did not display further improvements on SOR or NOR (Fig. 7, p > 0.4), and SOR and NOR memory performance did not differ between L12 and L20 mice receiving IGF2 (Fig. 7, p > 0.8). Considering an upper limit of 85% discrimination for object recognition tasks^40^, these results suggest that IGF2 and L20 do not influence memory in an additive manner.

## Discussion

Here we take a reverse translational approach to develop an animal model that provides novel insight into mechanisms underlying the positive effects of light on cognitive function. We have discovered that light-induced enhancement of recognition memory occurs in tandem with pronounced changes in hippocampal genetic programs - namely alteration of molecular clock function, up-regulation of *Igf2*, and elimination of *Wt1* expression. Based on our collective results, we propose that light-induced changes in hippocampal clock function leads to the de-repression of IGF2, which primes the hippocampus to enhance the acquisition and consolidation of long-term recognition memory. These results reveal new ways in which light regulates hippocampus-dependent memory formation and novel molecular mechanisms that may link the circadian clock to neuronal plasticity.

Clinical studies demonstrate that light can produce potent effects on cognitive function. Similarly, the effect of long days seen here is pronounced and consistent, with photoperiodic enhancement of spatial recognition memory in each of three experimental replicates. Previous work has established that object recognition protocols with a single 5-min training session often produce chance performance in mice tested after 24 h, with higher rates of discrimination typically requiring longer or multiple training sessions^40^. Consistent with the challenging nature of the training-testing regimen adopted here, mice held under standard lighting conditions failed to display successful discrimination when tested 24 h later. In contrast, mice exposed to long days consistently displayed 60–70% discrimination even though their behavior during training did not differ from control mice. This result is distinct from other models where cognitive deficits arise after exposure to non-naturalistic light manipulations (e.g., simulated jetlag, exposure to intermittent light pulses or above-threshold illumination throughout the night)^25, 41^. But our findings are consistent with previous work indicating that decreased intensity or length of daytime illumination produce affective and/or cognitive deficits in nocturnal and diurnal rodents^42, 43^. Indeed, enhanced recognition memory under long day photoperiods may reflect seasonal changes in cognition that provide an ecological advantage.

Although the amount of light exposure differed between L12 and L20 mice, behavioral and molecular data indicate that these groups were matched on the biological time of testing. Specifically, our previous work investigating locomotor and sleep rhythms indicate that these rhythms are similarly phased in L12 and L20 mice relative to the time of lights-off under each photoperiod^29, 30^. Consistent with these important behavioral indices, gene expression rhythms in the hippocampus indicate that L12 and L20 groups were tested at similar phases (present study, see also ref. 30). Thus, an important strength of the current work is that the effects of light exposure are assessed while controlling for biological phase. Importantly, the pattern of L20-induced enhancement of spatial recognition memory was evident at two different times of day, further indication that this effect is not merely due to a difference in the time of training and testing. In addition, we find that photoperiodic enhancement of recognition memory requires melanopsin signaling, but it remains possible that the classic image-forming visual system could contribute to this phenomenon. Of note, recent work suggests that both the classic and circadian visual system serve to encode the level of illumination present at the time of testing for recognition memory^44^. Our results complement this work by demonstrating that melanopsin signaling is necessary for encoding the effects of light exposure prior to testing. One issue for future work is to assess the role of the image-forming visual system and determine the ontogeny of light-induced enhancement of recognition memory. Studies addressing these issues are expected to provide insight into potential clinical applications.

It is well established that the circadian clock regulates cognitive function across the day^45^, but the specific molecular mechanisms connecting circadian function to neuroplasticity have remained unclear^35^. Circadian regulation of hippocampal function is associated with daily modulation of MAPK activity^46, 47^, but how the molecular clock regulates MAPK signaling or synaptic plasticity has not been defined. Here we reveal that long days change hippocampal clock function and elevate daytime expression of IGF2, which is known to be a critical growth factor regulating hippocampal-dependent memory. Up-regulation of *Igf2* occurred in tandem with suppressed transcription of its repressor *Wt1*, which we demonstrate is expressed rhythmically with peak expression occurring prior to the time that *Igf2* levels start to fall in the hippocampus. The rhythmic profile of *Wt1*, together with its conserved upstream regulatory sequences, suggests that *Wt1* is a clock-controlled gene regulated directly by CLOCK/BMAL1 and/or ROR/REV-ERBα. These results suggest that the molecular clock directly gates WT1 expression, which would serve to indirectly regulate IGF2 expression over the day. The effects of light on memory in the current study are likely due to the alteration of hippocampal clock function that changes the daily regulation of IGF2. Because IGF2 signaling has been linked to MAPK signaling^48, 49^, this highlights new areas for future research investigating the molecular links between circadian and cognitive function.

This study provides an in-depth investigation of the hippocampal clock, which expands on previous work^50–52^. Although we did not examine intrinsic rhythms of hippocampal transcripts, which would require *in vitro* analyses, daily rhythms in molecular function under entrained conditions are expected to have the most translational relevance. We find that exposure to long days altered core clock function in the hippocampus, but it is worth noting that the cognitive phenotype of L20 mice is distinct from the deficits seen in most clock gene knockout and mutant mice^25^. Although deficits are typically observed in studies using clock gene knockouts, there are two main exceptions. First, *Bmal1-/-* mice display a similar phenotype to L20 mice, with enhanced recognition and impaired fear conditioning^53^. The similarity of results with *Bmal1-/-* mice and L20 mice supports the conclusion that light-induced memory enhancement reflects changes in core clock function. In addition, *Dec1/2* knockout mice display enhancement of memory formation associated with cortical function, but not hippocampal function, with increased *Igf2* expression in the cortex but not the hippocampus^54^. Although the effect of *Dec1/2* knockout on cognitive function is thought to occur via changes in sleep homeostasis^55^, we have shown in previous work that L20 does not increase the amount of sleep per day or disrupt the daily sleep:wake rhythm^29, 30^. Based on our collective results, we propose that light-induced abrogation of core clock function leads to the de-repression of IGF2 via lowered levels of WT1, which primes the hippocampus in a manner that enhances long-term recognition memory. This adds to a growing body of studies that suggest reducing or eliminating clock function can have beneficial effects depending on context^56, 57^. Further work exploring the implications of naturalistic and non-naturalistic changes in lighting conditions for cognitive processes is warranted. Given that *Wt1* may be regulated by circadian mechanisms in both mice and humans, a key question is whether the molecular clock also regulates IGF2 expression in the hippocampus in a light-sensitive manner in humans. While differences in temporal niche are important to consider, it is of interest that many fundamental properties of circadian function are similar in nocturnal and diurnal species (e.g., SCN electrical rhythms, photic resetting, molecular clockworks)^58^.

## Methods

### Mice and lighting conditions

Homozygous PER2::LUC knockin mice^59^, backcrossed onto a C57Bl/6 background, were bred and raised under a 24 h light:dark (LD) cycle with 12 h of light and 12 h of darkness (L12, LD12:12, lights off: 1800 CST). Melanopsin knockout mice (*Opn4* ^−/−^) and wild-type controls were likewise bred and raised under L12. Throughout life, ambient temperature was maintained at 22 ± 2 °C, and animals had *ad libitum* access to water and food (Teklad Rodent Diet #8604). At 9-14 weeks of age, male mice of each genotype were transferred to individual cages. At this time, mice were either maintained under L12 or re-entrained to a 24 h light:dark cycle with 20 h of light (L20, LD20:4, lights off at 1800 CST). For both photoperiodic conditions, photophase light intensity was measured at 1100 ± 200 lux at the level of the cage floor. Cages were changed once every two weeks 30–60 min before the time of lights-off in each photoperiod. Mice remained under these photoperiodic conditions for at least 4 weeks prior to behavioral testing. Behavioral tests detailed below were conducted either during the light phase under low light conditions (~3 lux) or during the dark phase using dim red safe lights (~1 lux). All procedures were conducted according to the NIH Guide for the Care and Use of Animals and were approved by the Institutional Animal Care and Use Committees at Marquette University.

### Object Recognition Tests

L12 and L20 mice were tested for spatial object recognition (SOR) and novel object recognition (NOR) either in the light 6 h before lights-off (daytime testing) or in the dark starting immediately after lights-off (nighttime testing). Handling: Prior to testing, mice were handled for 2 min for 7 consecutive days at the time of eventual testing. Habituation: On the following 7 days, the mice were habituated to the testing apparatus by placing them for 5 min into a rectangular arena (57 × 35 × 20 cm box placed above a 7 × 7 cm grid). The arena was cleaned with 70% EtOH after each mouse. Mice remained in the behavioral suite for 1 h before and after habituation. Habituation sessions were videotaped using a high definition webcam mounted above the arena. The number of grid crossings completed by each mouse was counted to quantify activity levels and anxiety on each day of habituation. Training: For training, mice were placed into the arena along with two identical objects (clear glass cylinders) that were positioned along the back wall and secured to the floor. Mice were left to explore the objects for 5 min. As with habituation, the arena and the objects were cleaned between mice with 70% EtOH, mice were acclimated for 1 h before and after testing, and training sessions were videotaped. Testing: To test recognition memory, mice were placed into the arena along with two objects for 5 min either 2 h or 24 h after training to test short term memory (STM) and long-term memory (LTM), respectively. For SOR, both objects were identical to testing, but one was displaced to a new location. For NOR, one of the objects was replaced with a new, unfamiliar object (plastic yellow block). For both SOR and NOR, the left or right position of the altered object was counterbalanced across mice. As with habituation, the arena and objects were cleaned with 70% EtOH, mice remained in the suite for 1 h before and after testing, and training sessions were videotaped.

### IGF2 Injections

Recombinant mouse IGF2 (30 μg/kg, R&D Catalog#792-MG) was dissolved in 0.1% bovine serum albumin-phosphate-buffered saline. This dose was selected due to its ability to enhance hippocampus-dependent memory without adverse effects on physical, behavioral, and sensorimotor behavior^36^. On the day of SOR/NOR training, mice received subcutaneous IGF2 injections 20 min prior to being placed into the arena for daytime training (i.e., 6 h before lights-off). To control for the non-specific effects of injections, a saline vehicle was provided to separate groups of L12 and L20 mice. No injections were given on the following day for SOR/NOR testing, which occurred during the daytime 6 h before lights-off. All other aspects of the protocol were identical to that described above, with the exception that all mice were habituated to injections prior to training using saline injections provided on days of handling and habituation, starting on the third day of handling.

### Contextual Fear Conditioning

To test whether effects of light generalized to other forms of hippocampus-dependent memory, mice were tested for contextual fear conditioning (CFC) during the daytime (i.e., 6 h before lights-off). For habituation, mice were transported to the behavioral suite and handled for 2 min on 7 consecutive days. For training and testing, mice remained in the behavioral suite for 1 h before and after experimental procedures. For CFC training, following a 2-min baseline, each mouse received a series of 3 unsignaled 2-sec, 0.7 mA foot shocks separated by 1 min. Foot shocks were delivered using an electric shock circuit board (Med Associates, Inc. ENV-005-02A Rev. 1.0) and T/T Interface Cabinet Shock Generator (SG-6080C) in a conditioning chamber (Med Associates, Inc. Modular Test Chamber model ENV-008-FC, 30.5 cm × 24.1 cm × 21.0 cm) housed within a sound-attenuating box (Med Associates, Inc. Model ENV-022MD, 63.5 cm × 41.9 cm × 39.4 cm). A fan provided white noise and chambers were illuminated with a white house light (<1 lux intensity). For CFC testing, mice were returned 24 h later to the same conditioning chamber (context A) for 3 minutes with no foot shock. 24 h later, mice were placed in a novel chamber (context B) for 3 minutes with no foot shock. Context B differed from context A by red illumination, peppermint scent, a solid floor, and patterned walls. Behavior during CFC was recorded using wide-angle micro cameras positioned on the back wall of the sound-attenuating box.

### Mood Tests

Novelty-induced locomotor activity levels: During habituation to the object testing arena, general levels of locomotion were quantified using the number of grid crossings performed by each mouse. Porsolt Forced Swim Test (FST): Mice were tested during the daytime (6 h before lights-off) or nighttime (at the time of lights-off) by placing each mouse in a cylindrical tank (20 cm) filled with tap water (23–25 °C, 15 cm depth) and positioned below a high definition webcam. After 10 min, mice were retrieved, gently dried with a cotton towel and returned to their home cage. The tank was emptied, cleaned with 70% EtOH, and refilled for each mouse. 24 h later, mice were placed back into the filled tank for 5 min and swimming behavior was videotaped. Sucrose Preference Test (SPT): Mice were habituated to 2 bottles of regular water for 2 days. For the following 2 days, one bottle was switched out with 2% sucrose water, counterbalanced for side across each day. Bottles were placed 1 h before lights-off and the fluid remaining in each bottle was measured the following morning.

### Real Time quantitative PCR

Brains were collected from L12 and L20 mice not exposed to behavioral testing at timepoints spanning each photoperiod (n = 3/timepoint/photoperiod). After collection, brains were immediately fixed in 4% paraformaldehyde for 6 h, cryoprotected in 20% sucrose solution for 4 days, and then sectioned on a cryostat at 100μm in the coronal plane. The dorsal hippocampus was excised by hand with a scalpel from three consecutive slices. Tissue samples were homogenized, digested overnight at 55 °C in a nuclei lysis buffer supplemented with 50 μl of proteinase K (20 mg/ml). Total RNA was purified with TRIzol, mixed with chloroform, and then centrifuged for 15 min. RNA was precipitated with 70% isopropanol and centrifuged for 15 min. Resulting pellets were washed with 75% ethanol and centrifuged for 15 min. Pellets were then dried, re-suspended with 10 μl RNAase DNAase free dH20. RNA concentration and purity was quantified with 260/280 nm absorbance ratios measured with a Nanodrop 1000 spectrophotometer (Thermo Scientific, Wilmington, DE). First strand cDNA synthesis was achieved with reverse transcription using a high-capacity cDNA reverse transcription kit (Applied Biosystems, Cat#4368813). RNA and cDNA was stored at −20 °C. RT-qPCR assays were performed with an Applied Biosystems Step One Real-Time PCR System using Sso Advanced SYBR Green Supermix (Cat#172-5261). The thermal cycling program was set for 95 °C for 15 sec, 60 °C for 30 sec, and 72 °C for 45 sec for 40 cycles, followed by melt curve analysis using a 0.5 °C 5 sec stepwise gradient from 60 °C to 95 °C. Primer sequences are described in Table S2. *P0* was selected as the reference gene (NM_007475.5; Forward: CCGCCTGGTTCTCCTATAAAAGGCA, Reverse: CGATGTCACTCCAACGAGGACGC) based on its stable expression across the circadian cycle relative to four other commonly used genes (*βactin*, *18 S*, *cyclophilin*, *gapdh*). Cycle thresholds were analyzed with the ΔΔCT method by normalizing to the average value of expression under L12 collapsed across timepoints.

### Immunohistochemistry

To test the extent to which light altered IGF2 expression, brains were collected during the daytime before SOR training (i.e., 6 h before lights-off), 2 h after SOR training, or 20 h after SOR training from both L12 and L20 mice (n = 8/timepoint/photoperiod). To evaluate training-induced changes in IGF2, brains were collected from naïve L12 and L20 mice at the time equivalent to 20 h after SOR training (n = 8/photoperiod). After collection, brains were immediately post fixed in 4% paraformaldehyde overnight, cryoprotected in 20% sucrose solution for 4 days, and then sectioned on a cryostat (40 μm). Slices containing the hippocampus were collected and stored in cryoprotectant at −20 °C. Free-floating slices were washed in 0.1 M phosphate-buffered solution (PBS), and then incubated for 48 h at 4 °C with primary antibodies (Rabbit anti-IFG2, 1:500 as in ref. 37). Slices were then washed in PBS before 2 h incubation at room temperature with secondary antibodies (Jackson ImmunoResearch, Alexa Flour 488), washed with PBS, and mounted using Promount anti-fade gold reagent. Fluorescence images were obtained with a Nikon Eclipse 80i microscope using identical settings for all samples. IGF2 expression was calculated for each image by averaging the intensity values of five regions of interest manually positioned on each hippocampal region (Fig. 6A) and correcting for background staining.

### Data Analyses

Video records were scored off-line by two independent experimenters, and the inter-rater agreement was greater than r = 0.9. SOR/NOR: During object training and testing, the amount of time spent actively exploring each item was recorded. Active exploration was defined as the amount of time the mouse maintained nose-oriented close physical contact with each object (within 2 cm). Time spent on top of the object was not included. The discrimination ratio was calculated as the ratio of time spent with the unfamiliar object over the total time spent exploring both objects (i.e., Time spent with unfamiliar object/Time spent with both objects). Mood tests: During FST, we recorded the amount of time spent engaging in active escape behaviors (mobility) and passive behavior (immobility). Mobility was defined as any movements other than those required for floating. For SPT, preference was calculated as the amount of sucrose consumed overnight divided by the total fluid consumed overnight. CFC: Freezing was quantified as the lack of movement besides that required for breathing. Two experimenters hand scored the amount of freezing for mice in each group. Data was also analyzed with Freeze Scan CleverSys Inc., which automatically scores freezing by detecting pixel changes in the video. Software parameters were calibrated to match experimenter ratings. Statistical tests for daily rhythms in gene expression were analyzed with CircWave software^60^. Mean gene expression was calculated by calculating the average expression over the day or night phase of L12. Other statistical analyses were performed with JMP software (SAS Institute, Cary, NC).

## Electronic supplementary material

Supplementary Figures & Tables

## References

[1] Kripke DF, Mullaney DJ, Klauber MR, Risch SC, Gillin JC (1992). Controlled trial of bright light for nonseasonal major depressive disorders. Biol Psychiatry.

[2] Pail G (2011). Bright-light therapy in the treatment of mood disorders. Neuropsychobiology.

[3] Papadimitriou GN, Dikeos DG, Soldatos CR, Calabrese JR (2007). Non-pharmacological treatments in the management of rapid cycling bipolar disorder. J Affect Disord.

[4] Even C, Schroder CM, Friedman S, Rouillon F (2008). Efficacy of light therapy in nonseasonal depression: A systematic review. J Affect Disord.

[5] Bruin VM, Bittencourt LR, Tufik S (2012). Sleep-wake disturbances in Parkinson’s disease: Current evidence regarding diagnostic and therapeutic decisions. European neurology.

[6] Graf A (2001). The effects of light therapy on mini-mental state examination scores in demented patients. Biol Psychiatry.

[7] Riemersma-van der Lek RF (2008). Effect of bright light and melatonin on cognitive and noncognitive function in elderly residents of group care facilities: A randomized controlled trial. JAMA.

[8] Vandewalle G, Maquet P, Dijk DJ (2009). Light as a modulator of cognitive brain function. Trends in Cognitive Sciences.

[9] Forbes D, Blake CM, Thiessen EJ, Peacock S, Hawranik P (2014). Light therapy for improving cognition, activities of daily living, sleep, challenging behaviour, and psychiatric disturbances in dementia. Cochrane Database Syst Rev.

[10] Tuunainen, A., Kripke, D. F. & Endo, T. Light therapy for non-seasonal depression. *Cochrane Database Syst Rev*, CD004050 (2004). 10.1002/14651858.CD004050.pub2 PMC6669243 15106233

[11] Stephenson KM, Schroder CM, Bertschy G, Bourgin P (2012). Complex interaction of circadian and non-circadian effects of light on mood: Shedding new light on an old story. Sleep Med Rev.

[12] Mohawk JA, Green CB, Takahashi JS (2012). Central and peripheral circadian clocks in mammals. Annu Rev Neurosci.

[13] Buhr, E. D. & Takahashi, J. S. Molecular components of the mammalian circadian clock. *Handbook of Experimental Pharmacology*, 3-27 (2013). 10.1007/978-3-642-25950-0_1 PMC3762864 23604473

[14] Zhang EE, Kay SA (2010). Clocks not winding down: Unravelling circadian networks. Nature reviews. Molecular cell biology.

[15] McClung CA (2013). How might circadian rhythms control mood? Let me count the ways. Biol Psychiatry.

[16] Mattis J, Sehgal A (2016). Circadian rhythms, sleep, and disorders of aging. Trends in Endocrinology and Metabolism: TEM.

[17] Vandewalle G (2007). Wavelength-dependent modulation of brain responses to a working memory task by daytime light exposure. Cerebral Cortex.

[18] Vandewalle G (2010). Spectral quality of light modulates emotional brain responses in humans. Proc Natl Acad Sci.

[19] Schmidt TM, Chen SK, Hattar S (2011). Intrinsically photosensitive retinal ganglion cells: Many subtypes, diverse functions. Trends Neurosci.

[20] Li JZ (2013). Circadian patterns of gene expression in the human brain and disruption in major depressive disorder. Proc Natl Acad Sci.

[21] McCarthy MJ, Welsh DK (2012). Cellular circadian clocks in mood disorders. J Biol Rhythms.

[22] Johansson C (2003). Circadian clock-related polymorphisms in seasonal affective disorder and their relevance to diurnal preference. Neuropsychopharmacology.

[23] Musiek ES (2015). Circadian clock disruption in neurodegenerative diseases: Cause and effect?. Frontiers in Pharmacology.

[24] Kronfeld-Schor N, Einat H (2012). Circadian rhythms and depression: Human psychopathology and animal models. Neuropharmacology.

[25] Evans JA, Davidson AJ (2013). Health consequences of circadian disruption in humans and animal models. Prog Mol Biol Transl Sci.

[26] Terman M, Terman JS (2005). Light therapy for seasonal and nonseasonal depression: Efficacy, protocol, safety, and side effects. CNS Spectr.

[27] Nelson DE, Takahashi JS (1991). Sensitivity and integration in a visual pathway for circadian entrainment in the hamster (*Mesocricetus auratus*). J Physiol.

[28] Takahashi JS, DeCoursey PJ, Bauman L, Menaker M (1984). Spectral sensitivity of a novel photoreceptive system mediating entrainment of mammalian circadian rhythms. Nature.

[29] Evans JA, Leise TL, Castanon-Cervantes O, Davidson AJ (2013). Dynamic interactions mediated by nonredundant signaling mechanisms couple circadian clock neurons. Neuron.

[30] Evans JA (2015). Shell neurons of the master circadian clock coordinate the phase of tissue clocks throughout the brain and body. BMC Biology.

[31] Clark DA, Freifeld L, Clandininz TR (2013). Mapping and cracking sensorimotor circuits in genetic model organisms. Neuron.

[32] LeGates TA (2012). Aberrant light directly impairs mood and learning through melanopsin-expressing neurons. Nature.

[33] Wang LM (2009). Expression of the circadian clock gene *Period2* in the hippocampus: Possible implications for synaptic plasticity and learned behaviour. ASN Neuro.

[34] Lee E, Son H (2009). Adult hippocampal neurogenesis and related neurotrophic factors. BMB Reports.

[35] Smarr BL, Jennings KJ, Driscoll JR, Kriegsfeld LJ (2014). A time to remember: The role of circadian clocks in learning and memory. Behav Neurosci.

[36] Stern SA, Kohtz AS, Pollonini G, Alberini CM (2014). Enhancement of memories by systemic administration of insulin-like growth factor II. Neuropsychopharmacology.

[37] Agis-Balboa RC (2011). A hippocampal insulin-growth factor 2 pathway regulates the extinction of fear memories. EMBO J.

[38] Chen DY (2011). A critical role for IGF-II in memory consolidation and enhancement. Nature.

[39] Ueda HR (2002). A transcription factor response element for gene expression during circadian night. Nature.

[40] Antunes M, Biala G (2012). The novel object recognition memory: Neurobiology, test procedure, and its modifications. Cognitive Processing.

[41] Ikeno T, Yan L (2016). Chronic Light Exposure in the Middle of the Night Disturbs the Circadian System and Emotional Regulation. J Biol Rhythms.

[42] Pyter LM, Reader BF, Nelson RJ (2005). Short photoperiods impair spatial learning and alter hippocampal dendritic morphology in adult male white-footed mice (*Peromyscus leucopus*). J Neurosci.

[43] Ikeno T, Deats SP, Soler J, Lonstein JS, Yan L (2016). Decreased daytime illumination leads to anxiety-like behaviors and HPA axis dysregulation in the diurnal grass rat (*Arvicanthis niloticus)*. Behav Brain Res.

[44] Tam, S. K. *et al*. Modulation of recognition memory performance by light requires both melanopsin and classical photoreceptors. *Proc Biol Sci* **283** (2016). 10.1098/rspb.2016.2275 PMC5204172 28003454

[45] Schmidt C, Collette F, Cajochen C, Peigneux P (2007). A time to think: Circadian rhythms in human cognition. Cogn Neuropsychol.

[46] Eckel-Mahan KL (2008). Circadian oscillation of hippocampal MAPK activity and cAMP: Implications for memory persistence. Nat Neurosci.

[47] Philips GT, Ye X, Kopec AM, Carew TJ (2013). MAPK establishes a molecular context that defines effective training patterns for long-term memory formation. J Neurosci.

[48] Iwamoto T, Ouchi Y (2014). Emerging evidence of insulin-like growth factor 2 as a memory enhancer: a unique animal model of cognitive dysfunction with impaired adult neurogenesis. Reviews in the Neurosciences.

[49] Hu Q (2011). *Wt1* ablation and *Igf2* upregulation in mice result in Wilms tumors with elevated ERK1/2 phosphorylation. J Clin Invest.

[50] Ikeno T, Weil ZM, Nelson RJ (2013). Photoperiod affects the diurnal rhythm of hippocampal neuronal morphology of siberian hamsters. Chronobiol Int.

[51] Jilg A (2010). Temporal dynamics of mouse hippocampal clock gene expression support memory processing. Hippocampus.

[52] Valnegri P (2011). A circadian clock in hippocampus is regulated by interaction between oligophrenin-1 and Rev-erbalpha. Nat Neurosci.

[53] Wardlaw SM, Phan TX, Saraf A, Chen X, Storm DR (2014). Genetic disruption of the core circadian clock impairs hippocampus-dependent memory. Learn Mem.

[54] Shahmoradi A, Radyushkin K, Rossner MJ (2015). Enhanced memory consolidation in mice lacking the circadian modulators Sharp1 and-2 caused by elevated Igf2 signaling in the cortex. Proc Natl Acad Sci.

[55] Baier PC (2014). Mice lacking the circadian modulators SHARP1 and SHARP2 display altered sleep and mixed state endophenotypes of psychiatric disorders. PLoS One.

[56] Fernandez F (2014). Dysrhythmia in the suprachiasmatic nucleus inhibits memory processing. Science.

[57] Martino TA (2008). Circadian rhythm disorganization produces profound cardiovascular and renal disease in hamsters. Am J Physiol.

[58] Challet E (2007). Minireview: Entrainment of the suprachiasmatic clockwork in diurnal and nocturnal mammals. Endocrinology.

[59] Yoo SH (2004). PERIOD2::LUCIFERASE real-time reporting of circadian dynamics reveals persistent circadian oscillations in mouse peripheral tissues. Proc Natl Acad Sci.

[60] Oster H, Damerow S, Hut RA, Eichele G (2006). Transcriptional profiling in the adrenal gland reveals circadian regulation of hormone biosynthesis genes and nucleosome assembly genes. J Biol Rhythms.

