# Supplementary material for: Long days enhance recognition memory and increase insulin-like growth factor 2 in the hippocampus
Source: Sci Rep. 2017 Jun 20;7:3925. doi: 10.1038/s41598-017-03896-2 (PMC5478617; doi:10.1038/s41598-017-03896-2)
Supplement: Supplementary file 1 — Supplementary Figures & Tables [file 41598_2017_3896_MOESM1_ESM.pdf]

**Long days enhance recognition memory**
**and increase insulin-like growth factor 2 in the hippocampus**

Adriano Dellapolla, Ian Kloehn, Harshida Pancholi, Ben Callif, David Wertz, Kayla E. Rohr,
Matthew M. Hurley, Kimberly M. Baker, Samer Hattar, Marieke R. Gilmartin, Jennifer A. Evans

**Supplemental Tables**

**Table S1. Light-induced changes in hippocampal transcriptional programs.**

| | Rhythmicity | | Amplitude | | Mean Expression | |
|---|---|---|---|---|---|---|
| | **LD12:12** | **LD20:4** | **LD12:12** | **LD20:4** | **LD12:12** | **LD20:4** |
| **Clock genes** | | | | | | |
| *Bmal1* | p < 0.05 | p < 0.05 | 2.6 ± 0.2 | 1.0 ± 0.1** | 1.4 ± 0.2 | 0.9 ± 0.1* |
| *Clock* | p < 0.05 | p = 0.80 | 1.7 ± 0.2 | 0.6 ± 0.1** | 1.3 ± 0.2 | 0.9 ± 0.1** |
| *Cry1* | p < 0.005 | p = 0.12 | 2.0 ± 0.2 | 0.6 ± 0.1** | 1.3 ± 0.2 | 1.0 ± 0.1 |
| *Cry2* | p < 0.005 | p = 0.13 | 1.6 ± 0.1 | 0.7 ± 0.1** | 1.2 ± 0.1 | 0.8 ± 0.1* |
| *Dec1* | p < 0.005 | p < 0.05 | 3.1 ± 0.2 | 0.8 ± 0.1** | 1.7 ± 0.2 | 0.7 ± 0.1** |
| *Dec2* | p < 0.005 | p = 0.12 | 2.5 ± 0.2 | 1.1 ± 0.1** | 1.5 ± 0.2 | 1.0 ± 0.1* |
| *Per1* | p < 0.005 | p < 0.005 | 2.4 ± 0.2 | 1.0 ± 0.1** | 1.3 ± 0.2 | 0.9 ± 0.1* |
| *Per2* | p < 0.005 | p < 0.005 | 3.6 ± 0.2 | 1.9 ± 0.1** | 1.6 ± 0.2 | 0.8 ± 0.1* |
| *Rev-erbα* | p < 0.005 | p < 0.005 | 1.2 ± 0.1 | 2.3 ± 0.2** | 1.3 ± 0.1 | 1.9 ± 0.2* |
| *RorA* | p < 0.05 | p < 0.05 | 1.7 ± 0.1 | 0.7 ± 0.1** | 1.2 ± 0.1 | 0.8 ± 0.1* |
| **Growth Factors** | | | | | | |
| *Bdnf* | p < 0.005 | p = 0.16 | 1.7 ± 0.1 | 0.6 ± 0.1** | 1.3 ± 0.1 | 0.9 ± 0.1* |
| *Igf1* | p < 0.01 | p = 0.22 | 2.2 ± 0.2 | 0.9 ± 0.1** | 1.4 ± 0.2 | 1.0 ± 0.1 |
| *Igf2* | p < 0.05 | p < 0.01 | 2.1 ± 0.2 | 1.5 ± 0.2* | 1.3 ± 0.2 | 1.8 ± 0.2* |
| *Insulin* | p < 0.05 | p = 0.50 | 2.2 ± 0.3 | 1.1 ± 0.2** | 1.4 ± 0.2 | 0.9 ± 0.2 |
| *Vegf-a* | p < 0.05 | p = 0.40 | 1.1 ± 0.1 | 1.0 ± 0.1 | 0.8 ± 0.1 | 0.6 ± 0.1 |
| *Vegf-b* | p < 0.05 | p = 0.13 | 1.3 ± 0.1 | 0.7 ± 0.1** | 1.2 ± 0.1 | 1.0 ± 0.1 |
| **IGF2 Regulators** | | | | | | |
| *Egr1* | p < 0.05 | p < 0.05 | 2.1 ± 0.3 | 2.1 ± 0.2 | 1.6 ± 0.3 | 1.2 ± 0.2 |
| *Wt1* | p < 0.05 | p = 0.78 | 4.7 ± 0.3 | 0.7 ± 0.1** | 1.6 ± 0.3 | 0.8 ± 0.1* |

* p < 0.05, ** p < 0.005

**Table S2. Primers used for qRT-PCR**

| Gene | Forward Primer Sequence | Reverse Primer Sequence | Product Size, bp | Gene Accession |
|---|---|---|---|---|
| *Bdnf* | GCCTTTGGAGCCTCCTCTAC | GCTGTGACCCACTCGCTAAT | 150 | NM_007540.4 |
| *Bmal1* | GGACTTCGCCTCTACCTGTTC | CGTTGTCTGGCTCATTGTCTT | 131 | NM_007489.4 |
| *C-ebp* | ATCGACTTCAGCCCCTACCT | GGCTCACGTAACCGTAGTCG | 151 | NM_009883.4 |
| *Clock* | CCCAGAGGGAGAACATTCAG | TGGCTCCTTTGGGTCTATTG | 148 | AF000998.1 |
| *Cry1* | CCTTATCTCCGCTTTGGTTG | CACAGGAGTTGCCCATAAAGA | 119 | NM_007771 |
| *Cry2* | CTCCTGCCGCCTCTTCTAC | CCTCCATTCGGTCAAACCT | 152 | NM_009963 |
| *Dec1* | AAAGCCGTGGACTTGAAAGA | CCTTCTCCAATTCACCTCCA | 166 | NM_011498.4 |
| *Dec2* | AAAAGGAGCTTGAAGCGAGA | AATGCCCCAGTGTTGTCAAT | 151 | NM_001271768.1 |
| *Egr1* | GAGGAAGTTTGCCAGGAGTG | TGGGTAGGAGGTAGCCACTG | 148 | NM_007913.5 |
| *Igf1* | ACCACAGCTGGACCAGAGAC | GCAACACTCATCCACAATGC | 153 | NM_010512 |
| *Igf2* | GACGACTTCCCCAGATACCC | CGTTTGGCCTCTCTGAACTC | 155 | NM_010514.3 |
| *Insulin* | GGACCCACAAGTGGAACAAC | GCTGGTAGAGGGAGCAGATG | 131 | NM_008386.3 |
| *P0* | CCGCCTGGTTCTCCTATAAAAGGCA | CGATGTCACTCCAACGAGGACGC | 78 | NM_007475 |
| *Per1* | TGAGGAGCCAGAGAGGAAAG | GCAGTGTAGGAGGAGGAGGA | 141 | NM_011065 |
| *Per2* | GAAAGCTGTCACCACCATAGAA | AACTCGCACTTCCTTTTCAGG | 186 | NM_011066 |
| *Rev-erbα* | AGAGATGCTGTGCGTTTTGGGCG | AGGTGGGATGTGGAGTAGGTGAGGT | 142 | NM_145434.3 |
| *RorA* | GAACACCTTGCCCAGAACAT | TGCCACATCACCTCTCTCTG | 143 | NM_013646.2 |
| *Vegf-a* | TGAGACCCTGGTGGACATCT | CTGCATGGTGATGTTGCTCT | 151 | NM_009505.2 |
| *Vegf-b* | CAGCCAATGTGAATGCAGAC | GGAGTGGGATGGATGATGTC | 153 | NM_011697.3 |
| *Wt1* | AGGTTTTCTCGCTCAGACCA | GCTGAAGGGCTTTTCACTTG | 159 | NM_144783.2 |

**Supplemental Figures**

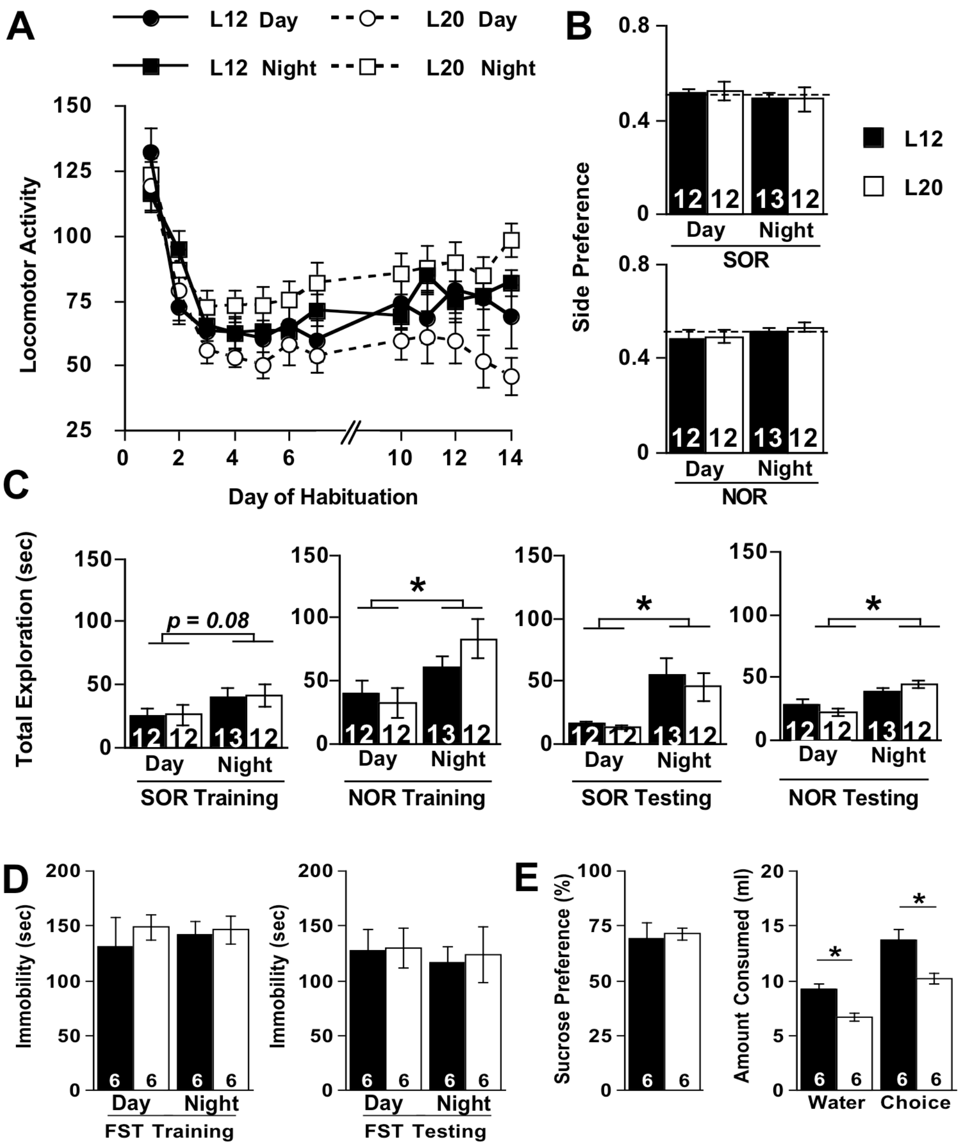


Figure S1. Simulated light therapy did not alter behavior exhibited during habituation, object training, or mood tests. **A.** Habituation of activity levels in groups trained 6 h before lights-off (Day) and after lights-off (Night) over consecutive days (Repeated Measures ANOVA, Change over Time: $p < 0.0001$) did not differ between photoperiodic groups at either time of day (Repeated Measures ANOVA, Photoperiod: $p > 0.2$; Photoperiod*Change over time: $p > 0.1$). **B.** During SOR and NOR training, L12 and L20 mice did not prefer the object on a specific side of the arena (One sample Student's t test, $p > 0.2$). **C.** Total exploration time was higher at Night than Day (Full Factorial ANOVA, Time of testing (TT): $p < 0.08$), but total exploration time did not differ between L12 and L20 mice (Full Factorial ANOVA, Photoperiod: $p > 0.5$; Photoperiod*TT: $p > 0.1$). **A-C.** Similar results were obtained for groups tested for short-term memory (data not shown). **D.** Neither time of day or photoperiod influenced the amount of time spent immobile on the Porsolt Forced Swim Test (Full Factorial ANOVA, TT: $p > 0.6$, Photoperiod: $p > 0.5$, Photoperiod*TT: $p > 0.6$). **E.** Photoperiod did not influence sucrose preference ($p > 0.7$), but did reduce the amount of fluid consumed on both the day of habituation to two bottles (water, $p < 0.005$) and sucrose preference testing (choice, $p < 0.05$). The reduction in fluid consumption is consistent with nocturnal drinking being curtailed by the shortened night of L20.

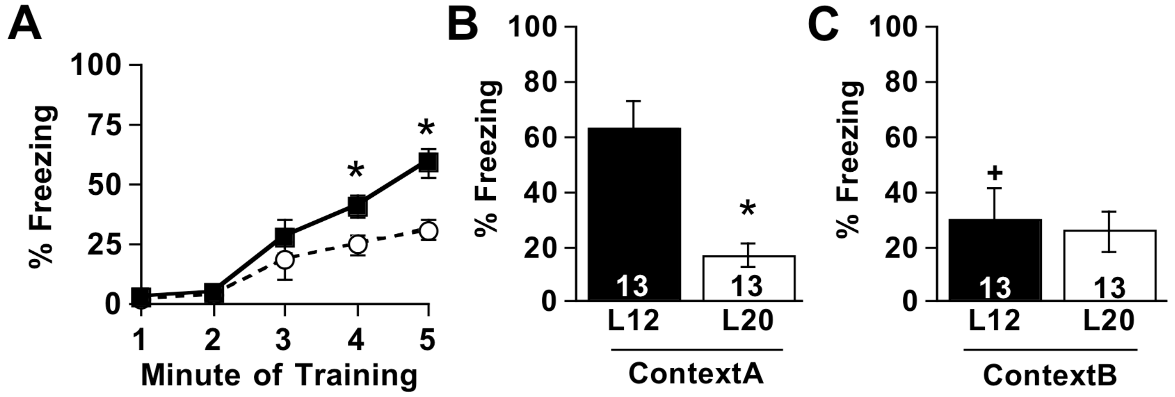


Figure S2. Simulated light therapy inhibits the acquisition of contextual fear conditioning. **A.** Acquisition curve for L12 and L20 mice during fear conditioning. *L12 versus L20, LS Means post doc comparisons, $p < 0.05$. B. L20 mice display reduced levels of freezing when exposed to Context A used for fear conditioning. *L12 versus L20, Student's t test, $p < 0.05$). **C.** In neutral Context B, freezing is reduced in L12 mice, but not L20 mice. *Context A versus Context B, $p < 0.05$). Number at the base of each bar indicates group sample size.

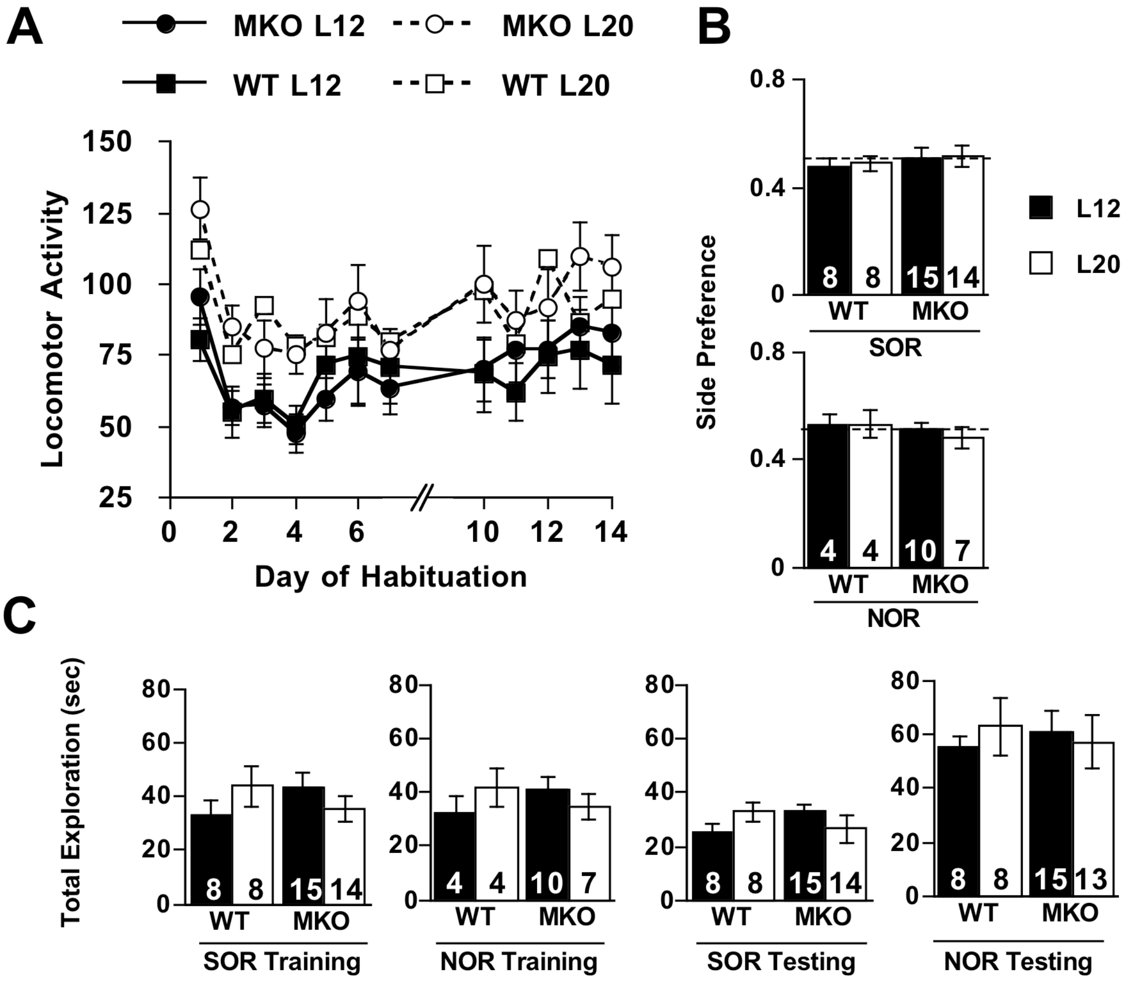


Figure S3. Behavior exhibited during habituation and object training was not altered by loss of melanopsin. **A-C.** Genotype did not affect locomotor activity levels during habituation (**A**, Repeated Measures ANOVA Time: $p < 0.005$, Genotype: $p > 0.6$, Photoperiod: $p < 0.05$, Genotype*Photoperiod: $p > 0.7$, Genotype*Time: $p > 0.5$, Photoperiod*Time: $p > 0.3$, Genotype*Photoperiod*Time: $p > 0.9$), side preference (**B,** Full Factorial ANOVA SOR $F(3,40) = 0.29$, $p > 0.8$; NOR: $F(3,21) = 0.65$, $p > 0.5$) or total exploration time (**C,** Full Factorial ANOVA SOR Training: $F(3,40) = 0.75$, $p > 0.5$; NOR Training: $F(3,21) = 1.51$, $p > 0.2$; SOR Testing: $F(3,40) = 1.08$, $p > 0.3$; NOR Testing: $F(3,39) = 1.55$, $p > 0.2$). Please note that NOR training videos for one cohort (n= 19) were lost due to computer failure, causing a decrease in sample size for these specific analyses. Also, one MKO L20 mouse had to be sacrificed due to a skin lesion in between SOR and NOR testing, causing a decrease in the sample size for this specific group for NOR analyses.

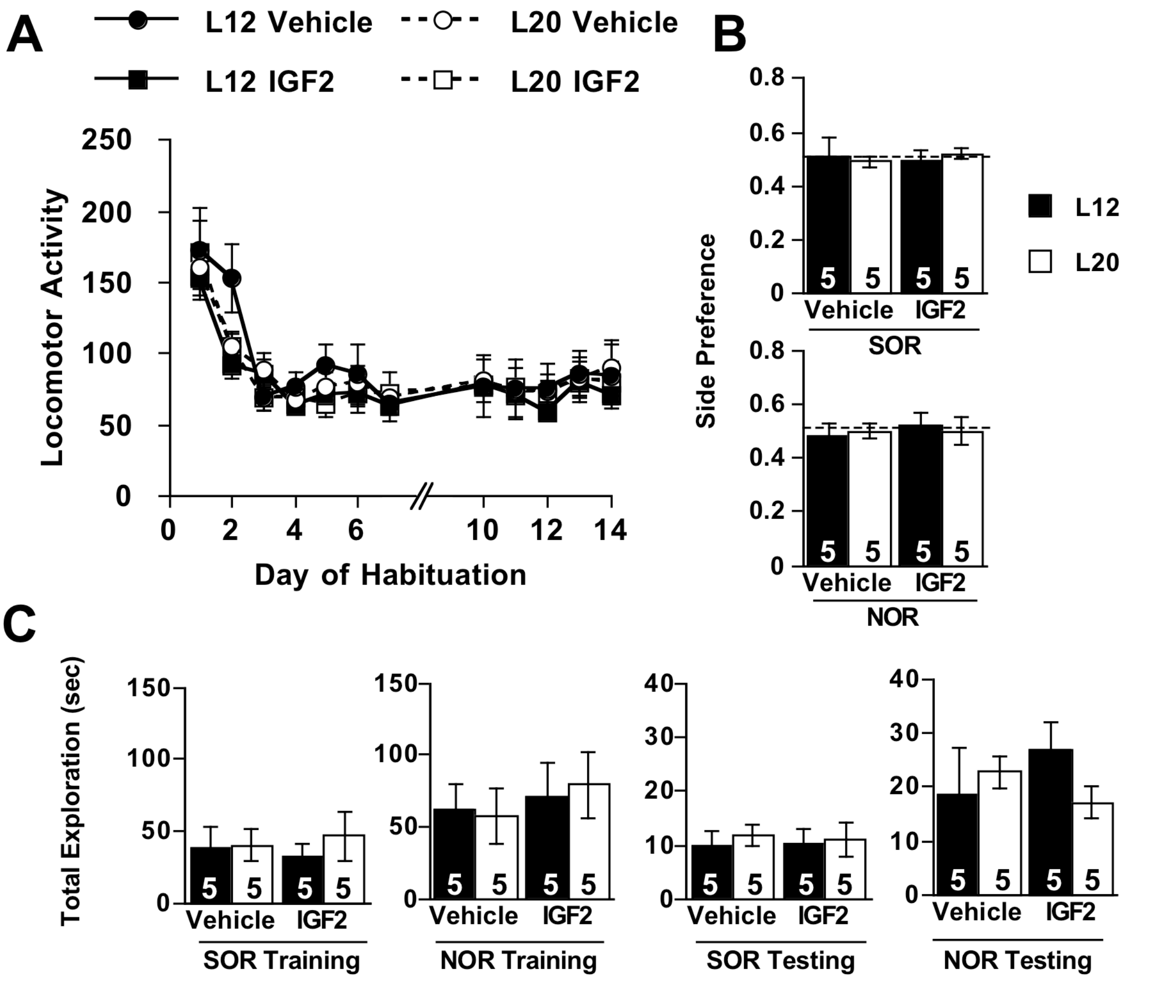


Figure S4. Behavior exhibited during habituation and object training was not altered by IGF2 injections. **A-C.** IGF2 injections did not affect locomotor activity levels during habituation (**A**, Repeated Measures ANOVA Time: $p < 0.005$, Injection: $p > 0.4$, Photoperiod: $p > 0.9$, Injection*Photoperiod: $p > 0.6$, Injection*Time: $p > 0.4$, Photoperiod*Time: $p > 0.7$, Injection*Photoperiod*Time: $p > 0.3$), side preference (**B,** Full Factorial ANOVA SOR: $F(3,16) = 0.87$, $p > 0.4$; NOR: $F(3,16) = 0.96$, $p > 0.4$), or total exploration time (**C,** Full Factorial ANOVA SOR Training: $F(3,16) = 0.21$, $p > 0.8$; NOR Training: $F(3,16) = 0.22$, $p > 0.8$; SOR Testing: $F(3,16) = 0.1$, $p > 0.9$; NOR Testing: $F(3,16) = 0.63$, $p > 0.6$).
